# CropGF: a comprehensive visual platform for crop gene family mining and analysis

**DOI:** 10.1093/database/baad051

**Published:** 2023-07-01

**Authors:** Jingtian Xu, Can Zhu, Minzeng Su, Sida Li, Haoyu Chao, Ming Chen

**Affiliations:** Department of Bioinformatics, College of Life Sciences, Zhejiang University, Hangzhou 310058, China; Department of Bioinformatics, College of Life Sciences, Zhejiang University, Hangzhou 310058, China; Department of Bioinformatics, College of Life Sciences, Zhejiang University, Hangzhou 310058, China; Department of Bioinformatics, College of Life Sciences, Zhejiang University, Hangzhou 310058, China; Department of Bioinformatics, College of Life Sciences, Zhejiang University, Hangzhou 310058, China; Department of Bioinformatics, College of Life Sciences, Zhejiang University, Hangzhou 310058, China

## Abstract

A gene family refers to a group of genes that share a common ancestry and encode proteins or RNA molecules with similar functions or structural features. Gene families play a crucial role in determining the traits of plants and can be utilized to develop new crop varieties. Therefore, a comprehensive database of gene family is significant for gaining deep insight into crops. To address this need, we have developed CropGF (https://bis.zju.edu.cn/cropgf), a comprehensive visual platform that encompasses six important crops (rice, wheat, maize, barley, sorghum and foxtail millet) and one model plant (Arabidopsis), as well as genomics, transcriptomics and proteomics data for gene family mining and analysis, covering a total of 314 611 genes and 4399 types of domains. CropGF provides a versatile search system that allows for the identification of gene families and their members in a single crop or multiple crops. Users can customize their search based on gene family domains and/or homology using keywords or BLAST. To enhance usability, we have collected the corresponding ID information from various public databases for both genes and domains. Furthermore, CropGF comprises numerous downstream analysis modules, such as ka/ks analysis, phylogenetic tree construction, subcellular localization analysis and more. These visually-displayed modules provide intuitive insights into gene expression patterns, gene family expansion and functional relationships across different molecular levels and different species. We believe that CropGF will be a valuable resource for deep mining and analysis in future studies of crop gene families.

**Database URL** https://bis.zju.edu.cn/cropgf

## Introduction

Crops are the staple food for many people worldwide, and their evolution and diversity are shaped by a variety of factors, including genetic variation. One important aspect of genetic variation is the existence of gene families, which are composed of homologous genes that share a common ancestor and have two or more copies derived from gene duplication. By exploring the gene families that underpin the development of crops, we can gain a deeper understanding of their genetic copy and the processes that have shaped their evolution over time (1). Members of the same gene family are sometimes closely located to form a gene cluster. However, in most cases, they are distributed on different locations of the same chromosome or dispersed on different chromosomes (2). A large proportion of genes (up to 90% in eukaryotes) can translate multi-domain proteins, with most domains being highly conserved and related to protein folding and function. In contrast to the protein sequence, where in some families relatives have been detected sharing fewer than 5% identical residues, in many protein families at least 50% of the structure, mainly in the core of the protein, is highly conserved and can be used as a fingerprint to detect very distant relatives (3). Therefore, we can consider the members of a gene family from the perspectives of domains, gene function and homology. Classification of genes into families is a crucial step for researchers to understand gene interconnectivity, expansion and predict the functions of newly discovered genes in different crops.

Gene families also play a critical role in understanding the genetic basis of important traits such as grain yield, disease resistance and stress tolerance in crops. Evidence suggests that gene families are the master regulators for diverse biological processes (4). In addition, gene families can also help researchers predict the function of unknown genes. For example, in *Arabidopsis thaliana*, AGC protein kinases have been shown to be involved in lipid signaling pathways, auxin regulation and photosynthesis. Based on the identification of the AGC gene family in *Arabidopsis*, Jiang Y *et al.* (5) successfully identified the ACG gene family in rice and validated its important regulatory role in photosynthesis . Moreover, gene families can provide insights into plant evolution. Winter, K.-U. *et al.* (6) indicates that Gnetales and conifers are more closely related to each other than to angiosperms according to phylogenetic analyses of MADS-box genes. Therefore, gene family research is an important field in agriculture science and crop gene family databases can provide valuable resources for crop improvement and genetic research.

In recent years, many gene family databases have been constructed for various plant species, such as PlantRegMap (7) and PLAZA (8). They enable the search of gene families in many common plants, making it possible to explore the expansion of these gene families in plants. This is due to the rapid increase in the number of sequenced and annotated plant genomes, which provides data support for the construction of gene family databases for more species. Some databases, on the other hand, target individual species, such as BGFD (2) and MGFD (9), which provides comprehensive annotation and analysis of gene families for a single species. They include the integration of multiple omics data with gene family information, allowing researchers to explore gene expression patterns and functional relationships at different molecular levels.

Despite the availability of numerous gene family databases, many of these resources suffer from certain limitations. While databases that cover a broad range of species may provide useful information, they often lack the level of detailed analysis necessary to fully understand the genetic characteristics of individual species. This requires users to conduct additional downstream analysis, which can be time-consuming and may result in a loss of accuracy. Conversely, databases that focus on specific species may lack the connections and broader perspective needed to understand the evolutionary relationships and expansions of gene families across different species. Moreover, gene families in most existing gene family databases have fixed family members. With gene families sorted in advance, they limit the ability of users to select gene family members according to their own needs and understanding. This creates a challenge for researchers seeking to identify specific genes for further study.

To address these limitations, we present a user-friendly gene family database, namely CropGF (https://bis.zju.edu.cn/cropgf), that does not classify genes into gene families in advance and enables customization of gene family members. Our database includes rice, wheat, arabidopsis, maize, barley, sorghum and foxtail millet, 314 611 genes in total (Table 1), providing genome, transcriptome and proteome information for almost each genes. Visualization tools allow users to easily explore gene family data, facilitating crop research and breeding efforts. By providing users with the intuitive data and ability to customize gene family members, our database represents an important resource for researchers seeking to better understand the genetic characteristics of closely related crops, while also enabling the exploration of gene family relationships across species.

**Table 1.** Statistics of CropGF

Species	Number of chromosomes	Number of genes	Types of domains	Number of 3D structures	Number of tandems
*Arabidopsis thaliana*	5	27 624	4217	23 579	2429
*Hordeum vulgare*	7	35 106	3935	15 534	3328
*Oryza sativa*	12	35 767	4023	32 812	2086
*Setaria italica*	9	35 518	4157	35 008	3073
*Sorghum bicolor*	10	34 027	4176	33 518	3023
*Zea mays*	10	39 035	4103	32 104	1661
*Triticum aestivum*	21	107 534	4224	105 010	14 088

## Materials and methods

### Data sources

This database focuses on one model plant and six crops, including arabidopsis, rice, wheat, maize, barley, sorghum and foxtail millet. All proteome sequences and GFF annotations were obtained from the public database EnsemblPlants (http://plants.ensembl.org/index.html). The longest transcript proteins of each gene were selected for subsequent analysis. Chromosome length, gene location and gene structure information were obtained from the each GFF file. The R package biomaRt v3.8 (10) was used to obtain gene IDs, gene descriptions, synonyms and transcript numbers from different databases, including EnsemblPlants, UniProt (https://www.uniprot.org/) and NCBI’s Gene (https://www.ncbi.nlm.nih.gov/gene/). The hidden Markov models of domains were downloaded from the Pfam website (http://pfam-legacy.xfam.org/) and biomaRt was also used to search for corresponding domain IDs and descriptions from Pfam, SMART (https://smart.embl.de/), CDD (https://www.ncbi.nlm.nih.gov/Structure/cdd/cdd.shtml) and InterPro (https://www.ebi.ac.uk/interpro/).

### Physicochemical properties, 3D structure and subcellular localization

The physicochemical properties of all selected proteins, including molecular weight (MW), isoelectric point (pI), instability index (II) and grand average of hydropathicity (GRAVY) were predicted using the website ProtParam tool (https://web.expasy.org/protparam/) by utilizing the Python third-party library, requests v2.22. The subcellular localization of the selected proteins was predicted using the website Wolf Psort tool (https://wolfpsort.hgc.jp/) with the help of the Python third-party library, Selenium v3.141.The PDB files of the selected proteins were obtained from the AlphaFold Protein Structure Database (https://alphafold.com/) and were visualized by using Web3DMol (11).

### Identification of domains and homologous sequences

The protein sequences were analyzed for domain prediction using the Pfamscan v1.6 (12) with the E-value set at 0.001. All data processing and statistics were performed using in-house scripts. The fasta files of the proteins from the seven species were merged. The diamond v2.0.14 (13) was used to build index from the merged fasta file and search for homologous sequences with an E-value set at 1e-5 and identity greater than 30%.

### Gene expression analysis

The gene expression data were obtained from the GEO (https://www.ncbi.nlm.nih.gov/geo/), Plant Public RNA-seq Database (14) and PlantNexus (15). Then we select the high-quality samples with UniquelyMappedRate greater than 0.8 and organized the data for expression in different tissues or under different treatments. Finally, R package pheatmap v1.0.12 was used to generate clustering heatmap.

### Gene collinearity and KaKs calculation

MCScanX (16) was used with default parameters to identify collinear blocks and tandem duplicated genes among and within different species. The collinear blocks were visualized using Circos v0.69-8 (17) and SynVisio (18). We selected gene pairs of collinear blocks and tandem duplicated genes and used KaKs_Calculator 2.0 (19) to calculate the synonymous substitution rate (Ks), non-synonymous substitution rate (Ka), as well as Ka/Ks ratio. Visualization was completed through a scatter plot with Apache ECharts (https://echarts.apache.org).

### Phylogenetic analysis and motif prediction

Muscle v5.1 (20) was used to perform multiple sequence alignment of protein sequences. FastTree v2.1 (21) was used to construct a phylogenetic tree with default parameters, which was visualized using D3.js (https://d3js.org/). We used MEME v5.0.5 (22) to predict motifs with a maximum number of motifs set at 15 and E-value < 1e-40.

### Database construction

**Figure 1.**
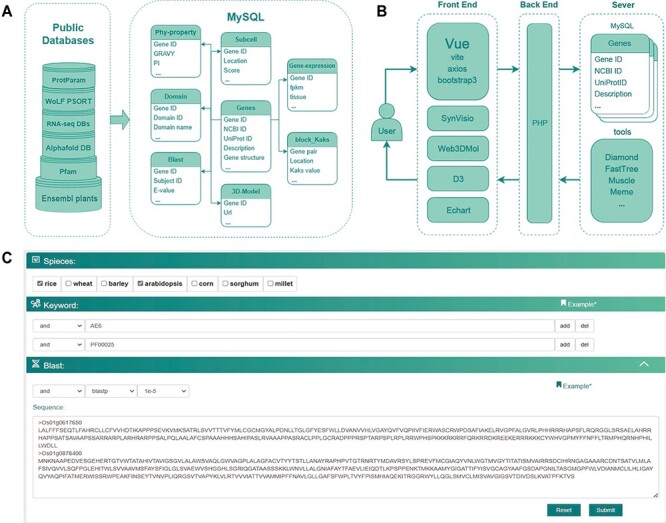
**The database construction and MySQL table structure utilized in this study.** (A) Public databases or analysis websites providing the data which stored in MySQL. Each cylinder in the left frame represents a different public database or analysis website, with the text on the cylinders denoting their respective names or abbreviations. Ensembl plants and Pfam are located at the bottom, with RNA-seq DBs and AlphaFold DB in the middle layer. RNA-seq DBs comprise GEO, Plant Public RNA-seq Database and PlantNexus, while AlphaFold DB represents the AlphaFold Protein Structure Database. Proparam and WoLF PSORT are positioned at the top. The right frame depicts the design of the MySQL tables, specifically Phy-property, Subcell, Gene-expression, Domain, Blast, 3D-Model, Block_Kaks and Genes. The arrows represent different tables linked through the Gene ID field. (B) The database construction relied on plug-in components, tools and frames. (C) Search module utilized together with options (AND, OR), combining keywords and blast in a certain way in accordance with specific research requirements.

CropGF is accessible for non-commercial use through the website. The server runs on the Linux system Ubuntu 20.04. The frontend was built using Vue 3 (https://cn.vuejs.org/). The visualizations were supported by ECharts.js and D3.js. The backend was supported by PHP 7.4(https://www.php.net/) and Nginx v1.14 (https://nginx.org/en/). The MySQL v8.0 database server was implemented for storing and managing genomics, transcriptomics and proteomics data (Figure 1).

## Usage and function

### Search

Our gene family database facilitates a flexible and expedient search system that allows users to retrieve gene family members based on domain and homology (Figure 1C). To initiate the search process, users must first select one or more species, and all subsequent searches will be conducted only within the chosen species. The database allows three search strategies for identifying potential gene family members: 1. Keyword search for gene family candidates that are homologous to a specific gene. The keywords can include gene IDs from different databases such as EnsemblPlants, UniProt or NCBI, as well as gene names, synonyms or descriptions. The database will search for genes matching the user’s criteria and return homologous genes as gene family candidates. 2. Keyword search for gene family candidates that contain a specific domain. The keywords can include the HMM ID and name of domains in the Pfam database. The database will search for proteins containing the specified domains and return corresponding genes as gene family candidates. 3. Blast search for gene family candidates. Users can submit sequences and arrange parameters for the blast search in the database, and the results will be returned as gene family candidates.

Users can add numerous keywords and set boolean operators (AND, OR) to combine the target genes in accordance with their requirements. For instance, by selecting barley as the species and submitting the sequence of the NB-ARC gene family member from *arabidopsis* for blast search, while specifying the boolean option as “AND” and searching for the keyword “PF00931” (the domain in the NB-ARC gene family), users can find the NB-ARC gene family in barley.

### Basic information

**Figure 2.**
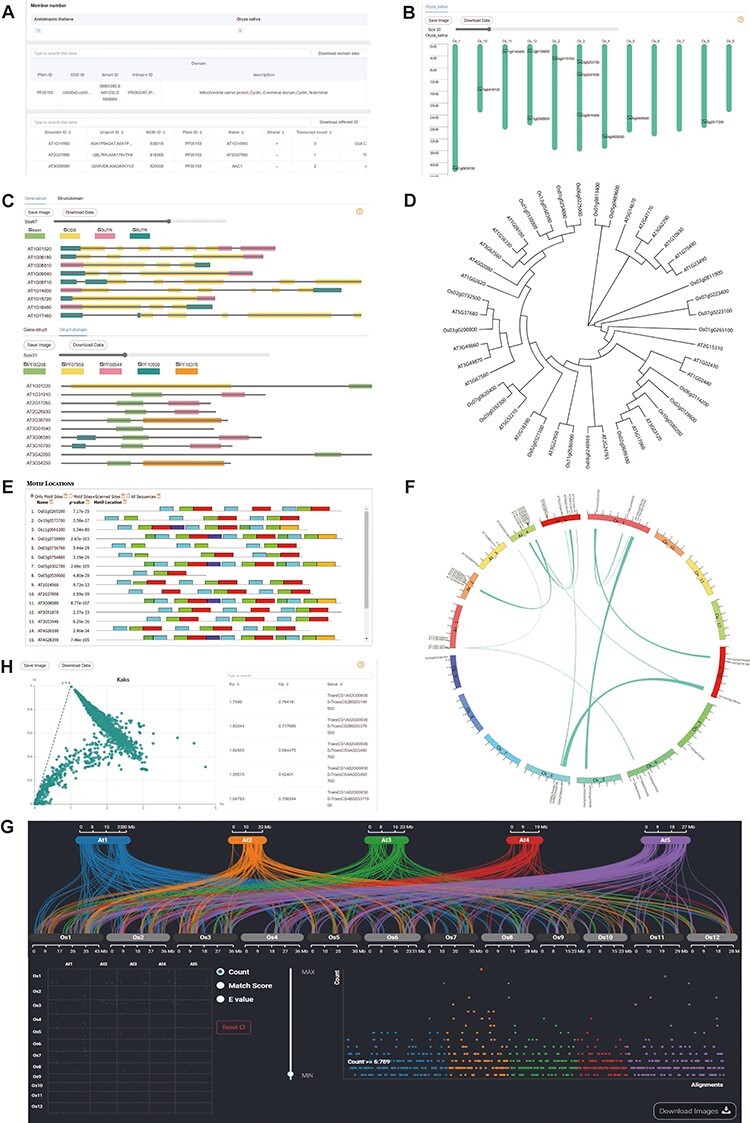
**Downstream analysis modules at genome level.** (A) Basic-info module. The top part indicates the number of family members in each species. The middle section gives information about the domains (if any) in the gene family. The bottom part lists the specific information about the family members. (B) Chromosome Location module with members on each chromosome. (C) Gene-Structure module with visualized structures on gene and domains on protein. (D) Tree module with phylogenetic tree. (E) Motif module with motif locations. (F) Collinerity module with visualized collinerity gene on circle diagram. (G) Sythi module with parallel link plot and dot plot of collinerity gene plot. (H) KaKs module, scatterplot on the left and search table on the right.

Database provides users with the number of gene family members found in different species user select. This information can be used as evidence of gene family expansion or origin in different species. Additionally, to provide users with more comprehensive information about the gene family across different databases, we list domain IDs and a brief description in different databases, including Pfam, SMART, CDD and InterPro. The module also displays the IDs in different databases (EnsemblPlants, UniProt and NCBI) and basic information of gene family members, which supports simple filtering (Figure 2A).

### Chromosome location and gene structure

The distribution of genes on chromosomes can reflect the expansion of the gene family. Therefore, we also visualized the chromosome location (Figure 2B). In addition, intron gain and loss are common phenomena in evolution, which increase the complexity of genomic organization (23). To examine the variation in evolution, each member’s gene structure and domains are visualized, which is provided a slider to zoom in on the length of gene units for users to observe more details (Figure 2C).

### Phylogenetic analysis and motif prediction

**Figure 3.**
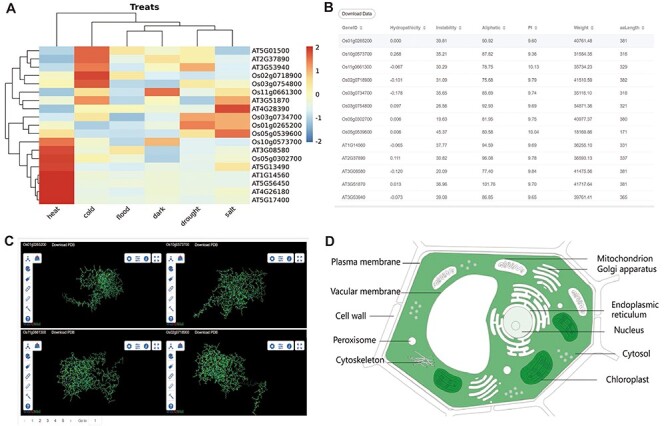
**The other part of downstream analysis modules about transcriptome and proteome.** (A) Expression module, gene expression in heat, cold, flood, dark, drought and salt conditions visualized by heatmap. (B) Physicochemical properties modules with PI, hydropathicity, Aliphatic, Instability, Weight and amino acids length. (C) 3D-Model module presenting four 3D structures at once. The ID and download are at the top of each structure. (D) Subcell modules with dark part meaning more genes localized there.

CropGF enables users to generate a phylogenetic tree in real-time based on the gene family of interest, which facilitates a comprehensive understanding of the expansion process of the gene family (Figure 2D). Additionally, CropGF provides motif prediction information, allowing users to identify conserved sites within each member of the gene family that can serve as a foundation for further research (Figure 3E).

### Gene collinearity and Kaks calculation

Gene collinearity analysis is a useful tool for detecting large-scale gene duplication events. The CropGF database can display collinear blocks of the gene family in the selected species range (Figure 2F, E). To investigate the impact of environmental selection on the gene family during evolution, CropGF also can identify tandem repeat or collinear paired genes within the gene family to calculate their Ka/Ks ratio, and generate a scatter plot (Figure 3G). This allows users to easily determine the extent of pressure on the gene family under environmental selection.

### Gene expression

Gene expression analysis is a crucial approach to investigate the functions of genes in various tissue development and treatment responses, as well as to explore the function of gene families. In order to visualize the expression of each member of the gene family in different tissues and stress environments, we have selected several important tissues and stress conditions, and utilized a clustering heatmap to display the gene expression level (FPKM) (Figure 3A).

### Physicochemical properties, 3D protein structure and subcellular localization

To facilitate users’ subsequent research and compare the mutations generated by each member of the gene family during evolution, the database displays the physical properties, 3D structure models and subcellular localization data of the longest transcript protein of each member of the gene family. Physicochemical properties are presented in the table (Figure 2B). Four 3D structures are shown at once for easy structural comparison in 3D-Model module (Figure 2C). In the Subcell module, the organelle of darker color means more genes are localized there (Figure 2D).

### Other cropGF systems

Home page includes a brief introduction of CropGF and species database contains. User can move the mouse over the picture of the plant to see simple information about the corresponding species. Help page introduces the usage of CropGF. Links pages have links of widely-used biological database or online analysis website. Download page provides some data in each species.

## Discussion

Here, we construct a gene family database for 1 model plant and 6 crops namely CropGF, which has several main applications. Firstly, it can be utilized to investigate the expansion of gene families. Researchers can conduct comparisons of the number of gene family members among different species and conduct an analysis of phylogenetic trees, gene collinearity, gene distribution on chromosomes and kaks to comprehend how genes have expanded. Additionally, through the examination of gene structures and motifs, researchers can infer the conserved blocks of gene families. Secondly, the database facilitates the cultivation and development of new crop varieties. Researchers can identify family members in other crops by utilizing known gene families and screen for essential genes via modules, including gene expression levels, subcellular localization and protein 3D structure, to improve crops. Thirdly, the database can be used to access information about gene family members. Users can retrieve comprehensive information regarding each gene family member, including protein 3D structure, domains, physicochemical properties. Users can also explore further detailed information using corresponding IDs in other databases.

Overall, CropGF is a comprehensive database that covers multiple significant crops and facilitates the exploration of crucial physiological traits of crops. The database is equipped with a flexible search system that permits users to use several search terms and select appropriate bool options for a more precise description of gene families, leading to the identification of more accurate family members. In addition, CropGF has a complete set of downstream analysis and a vast dataset that combines omics data with gene family data. This integration enables the downstream module to cover common gene family analysis methods and display them visually, allowing users to explore gene expression patterns and functional relationships at different molecular levels. The use and resources of the database are free and user-friendly, making it easy for people from different backgrounds to use.

However, the database does not include all important crops for human production. In addition, increasing cutting-edge biotechnologies, such as scRNA-seq and spatial transcriptomics, have been applied to various crops. It produces a multitude of data that is of great value to further integration into database. Thus, the kind of species will be expanded in our database and soybean, cotton and rape are given high priority. Meanwhile, we will actively collect these latest data and explore corresponding visualization solutions. Nevertheless, we still believe that CropGF will become a valuable resource for deep mining and analysis in future studies of crop gene families.

## Data Availability

The raw data can be found in the “Download” section. Downstream analysis data can be downloaded in each module, including text and images.
